# STRUCTURAL AGEISM AND PRODUCTIVE ACTIVITIES: IMPLICATIONS FOR HEALTH EQUITY RESEARCH

**DOI:** 10.1093/geroni/igae098.1020

**Published:** 2024-12-31

**Authors:** Ernest Gonzales, Cliff Whetung, Jane Lee, Stacey Gordon, Natalie Green

**Affiliations:** New York University, New York, United States; University of Minnesota, Duluth, Duluth, Minnesota, United States; University of Hawaiʻi at Mānoa, Honolulu, Hawaii, United States; New York University, New York, New York, United States; New York University, New York, New York, United States

## Abstract

Ageism is structural: hostility, incivility, and discrimination crosscut socio-ecological domains from intrapersonal beliefs and behaviors to interpersonal dynamics in everyday interactions within the workplace, neighborhood, and in health care settings. Ageism also intersects with minoritized identities that often result in experiences of racism and sexism, and elevates the risk for worse health outcomes. This presentation is informed by structural ageism in the productive aging framework (Morrow-Howell & Gonzales, 2023) and has three main goals: first, we apply an intersectional lens to examine the relationships of discrimination in the workplace, everyday interactions, and neighborhoods with health and retirement among a diverse sample (Hispanics, Blacks, and Whites) in the United States. This goal primarily focuses on employment contexts. The second goal is to reveal risk and protective factors that mediate the relationship between discrimination and cognitive health among Indigenous, Hispanic, Blacks, and Whites in the United States. Here, we examine the unique contribution of civic engagement. Finally, we will share our latest research on ageism in the family and health – with an emphasis on informal caregiving. Across these studies, productive activities are our organizing principle – employment, civic engagement, and caregiving – to examine risk and protective factors with health. Data primarily come from the Health and Retirement Study (2006-2020) with longitudinal analyses that informed goals 1 and 2 above; the third goal is informed by critical reflections in geriatric care management and social work practice. We conclude with implications for future research, namely recruiting diverse samples, experimental and intervention research.

